# Beyond need satisfaction: the role of psychological need frustration in sport injury anxiety among High-Intensity Functional Training participants

**DOI:** 10.3389/fpsyg.2026.1902192

**Published:** 2026-08-07

**Authors:** Umutcan Bozkuş, Zarife Pancar, Burak Karaca, Muhammed Kaan Darendeli, Mustafa Sencer Ulema, Nursel Üneşi

**Affiliations:** 1 Department of Physical Education and Sports, Institute of Health Sciences, Gaziantep University, Gaziantep, Türkiye; 2 Department of Physical Education and Sports, Faculty of Sports Science, Gaziantep University, Gaziantep, Türkiye

**Keywords:** exercise psychology, High-Intensity Functional Training, psychological need frustration, Self-Determination Theory, sport injury anxiety

## Abstract

**Purpose:**

This study aimed to investigate psychological need frustration as a predictor of sport injury anxiety among HIFT athletes, and to examine the roles of demographic and training-related variables in injury-related anxiety.

**Methods:**

A cross-sectional research design was employed. The sample consisted of 100 volunteer adult High-Intensity Functional Training participants from Türkiye (66 males, 34 females; age = 30.30 ± 6.75 years). Data were collected using the Sport Injury Anxiety Scale (SIAS) and the Basic Psychological Needs in Sport Scale (BPNSS). Independent-samples *t*-tests, one-way analyses of variance (ANOVA), Pearson correlation analyses, and standard multiple linear regression analyses were performed.

**Results:**

Overall sport injury anxiety was found to be below the midpoint of the scale (*M* = 2.23, SD = 0.59). Reinjury anxiety demonstrated the highest mean score (*M* = 3.02, SD = 1.09), whereas loss of social support anxiety showed the lowest mean score (*M* = 1.48, SD = 0.69). Correlation analyses revealed significant positive associations between sport injury anxiety and autonomy frustration (*r* = 0.32, *p* = 0.001), competence frustration (*r* = 0.29, *p* = 0.003), and relatedness frustration (*r* = 0.38, *p* < 0.001). In contrast, no significant relationships were observed between sport injury anxiety and any of the psychological need satisfaction dimensions (*p* > 0.05). Female participants reported significantly higher pain-related anxiety than male participants (*p* = 0.041, *d* = 0.44, women > men). Married participants demonstrated significantly lower overall injury anxiety than unmarried/widowed participants (*p* < 0.001, *d* = 0.83). Regression analyses identified age (*β* = −0.21, *p* = 0.037), sex (*β* = 0.26, *p* = 0.011), marital status (*β* = 0.24, *p* = 0.013), and relatedness frustration (*β* = 0.34, *p* = 0.017) as significant predictors of sport injury anxiety. The model explained 31.8% of the variance in sport injury anxiety (*R*^2^ = 0.318, *F*(7, 92) = 6.14, *p* < 0.001).

**Conclusion:**

These findings extend SDT to the sport injury anxiety domain by demonstrating that need frustration, particularly relatedness frustration, constitutes a significant and independent psychological risk factor for injury-related concerns in HIFT participants.

## Introduction

High-Intensity Functional Training (HIFT) is a training modality characterized by the combination of functional movements, resistance exercises, Olympic lifting derivatives, and metabolic conditioning performed at relatively high intensities (Hak et al., 2013). The primary objective of the program is the simultaneous development of multiple physical capacities, including strength, endurance, flexibility, coordination, agility, and cardiorespiratory fitness (Claudino et al., 2018). With thousands of facilities offering HIFT programs worldwide and participants representing a wide range of age groups, HIFT has become one of the most prominent components of contemporary fitness culture (Hak et al., 2013). Although regular participation in HIFT has been associated with improvements in aerobic capacity, muscular strength, and body composition (Kliszczewicz et al., 2014), its high-intensity and competitive nature also warrants closer examination of the psychological factors associated with participation (Davitt et al., 2014).

One of the psychological constructs that may influence athletes’ performance and participation experiences is sport injury anxiety. Sport injury anxiety refers to the cognitive and emotional responses athletes develop regarding the possibility of sustaining an injury, experiencing pain, losing performance capacity, or becoming disconnected from their social environment (Klimek et al., 2018). Accumulating evidence suggests that injury anxiety, alongside stress and athletic identity, constitutes a core psychological risk factor that may increase athletes’ vulnerability to both acute and overuse injuries (Johnson and Ivarsson, 2025). Subsequent research has expanded this framework by incorporating additional psychological variables such as self-confidence, psychological resilience, motivation, and coping skills, highlighting the multifaceted nature of injury-related psychological responses (Petrie et al., 2014).

In recent years, one of the most influential theoretical frameworks for understanding injury risk and injury-related anxiety in sport has been Self-Determination Theory (SDT). According to Ryan and Deci (2017), optimal psychological functioning and personal development depend on the satisfaction of three fundamental psychological needs: autonomy, competence, and relatedness. Autonomy refers to experiencing a sense of volition and self-endorsement in one’s actions, competence reflects perceptions of effectiveness and mastery in dealing with environmental demands, and relatedness represents the experience of meaningful and supportive interpersonal relationships (Ryan and Deci, 2017). Within sport settings, satisfaction of these needs has consistently been associated with greater motivation, performance, engagement, persistence, and psychological wellbeing (Sarı et al., 2022).

An important development in contemporary SDT research has been the growing recognition that psychological need frustration constitutes a distinct psychological process, rather than simply the absence of need satisfaction. Bartholomew et al. (2011) argued that the active thwarting of individuals’ psychological needs produces outcomes that differ substantially from merely failing to satisfy those needs. Accumulating evidence has linked psychological need frustration to anxiety, burnout, stress, negative affect, and reduced psychological wellbeing (Bartholomew et al., 2011; Vansteenkiste and Ryan, 2013). Despite this growing body of literature, the relationship between psychological need frustration and sport injury anxiety remains largely unexplored.

A review of the existing literature indicates that HIFT research has primarily focused on injury epidemiology (Weisenthal et al., 2014; Montalvo et al., 2017), physical performance outcomes (Claudino et al., 2018), exercise motivation (Partridge et al., 2014), and quality of life indicators. Despite the growing body of research on HIFT participation, limited attention has been devoted to understanding the psychological mechanisms underlying injury-related concerns. In particular, evidence regarding the association between psychological need frustration and sport injury anxiety among HIFT athletes remains scarce. This gap is noteworthy because it lies at the intersection of two well-established research domains: Self-Determination Theory and the psychology of sport injuries.

In addition to psychological needs, demographic and training-related characteristics may also contribute to injury-related anxiety. Previous studies have reported inconsistent findings regarding sex differences, with some investigations suggesting that female athletes experience higher levels of pain-related and reinjury anxiety (Correia and Rosado, 2019), whereas others have found no significant sex-based differences (Edison et al., 2021). Similarly, marital status may influence injury anxiety through the availability of social support resources (Brewer and Cornelius, 2001). Training frequency, training experience, and duration of sport participation have also been proposed as factors that may shape both psychological need experiences and perceptions of injury risk. Therefore, the present study aimed to investigate psychological need frustration as a predictor of sport injury anxiety among HIFT participation, while also examining the relative contributions of demographic and training-related variables to injury-related concerns. By extending Self-Determination Theory to the context of sport injury anxiety, this study seeks to contribute to a deeper understanding of the psychological mechanisms underlying injury-related concerns in HIFT settings.

Based on the theoretical framework of Self-Determination Theory and previous evidence linking need frustration to maladaptive psychological outcomes (Bartholomew et al., 2011; Vansteenkiste and Ryan, 2013), it was hypothesized that psychological need frustration would be positively associated with sport injury anxiety, whereas psychological need satisfaction was expected to demonstrate weaker or non-significant associations. More specifically, given the highly social and community-oriented nature of HIFT training, relatedness frustration was anticipated to emerge as the strongest psychological predictor of injury-related anxiety among the three need frustration dimensions.

## Methods

### Study design

This study employed a cross-sectional observational design to examine the relationships between sport injury anxiety and basic psychological need satisfaction and frustration among HIFT participants. A quantitative research approach was adopted, and data were collected using validated self-report questionnaires. The study was conducted in accordance with the principles outlined in the Declaration of Helsinki. The study protocol was approved by the Social and Human Sciences Local Ethics Committee of Gaziantep University (Approval No. 67; Date: 06 June 2023). Participation was voluntary, and all participants provided written informed consent prior to enrolment.

### Participants

Participants were recruited from private fitness centers offering High-Intensity Functional Training (HIFT) programs in Gaziantep, Türkiye, between April and September 2023. Eligibility criteria included: (a) being at least 18 years of age, (b) regular participation in HIFT programs for a minimum of 3 months, and (c) providing written informed consent. Individuals who were not actively engaged in HIFT programs at the time of data collection or who submitted incomplete questionnaires were excluded from the study. A total of 100 recreational HIFT participants met the inclusion criteria and were included in the final analyses. The sample consisted of 66 men (66%) and 34 women (34%), with a mean age of 30.30 ± 6.75 years. Participants reported a mean body mass index (BMI) of 24.84 ± 3.73 kg/m^2^. Regarding training experience, 38% of the participants had been involved in HIFT programs for 1 year or less, 25% for 2 years, 13% for 3 years, and 24% for 4 years or more. Regarding weekly training frequency, 15% of the participants trained twice per week, 32% three times per week, 25% four times per week, and 28% more than four times per week. To evaluate sample adequacy, a *post hoc* statistical power analysis was conducted using G*Power version 3.1 (Faul et al., 2007). Based on the observed explained variance (*R*^2^ = 0.318) and the seven predictors included in the regression model, the calculated effect size was large (*f*^2^ = 0.47), yielding statistical power greater than 0.90 at an alpha level of 0.05. Although post hoc power analyses should be interpreted cautiously, these findings suggest that the sample size was sufficient to detect the observed effects with an acceptable level of statistical precision.

## Measures

### Sport injury anxiety scale

Sport injury anxiety was assessed using the Sport Injury Anxiety Scale (SIAS), originally developed by Rex and Metzler (2016) and adapted into Turkish by Caz et al. (2019). The scale consists of 19 items distributed across six dimensions: loss of athletic ability (LA), being perceived as weak (PW), pain anxiety (PA), disappointment anxiety (DA), loss of social support (LSS), and reinjury anxiety (RA). Responses are recorded on a 5-point Likert scale ranging from 1 (strongly disagree) to 5 (strongly agree), with higher scores indicating greater injury-related anxiety. In the present study, internal consistency coefficients (Cronbach’s *α*) for the SIAS subscales were as follows: LA = 0.66, PW = 0.88, PA = 0.87, DA = 0.97, LSS = 0.96, RA = 0.80. The relatively lower *α* for the LA subscale (0.66) is consistent with its three-item format and has been reported in previous adaptations of the scale.

### Basic psychological needs in sport scale

Basic psychological need satisfaction and frustration were assessed using the Basic Psychological Needs in Sport Scale (BPNSS), developed by Bhavsar et al. (2020) and adapted into Turkish by Bülbül and Akyol (2020). The Turkish version consists of 28 items assessing six dimensions: autonomy satisfaction (AutoS), autonomy frustration (AutoF), competence satisfaction (CompS), competence frustration (CompF), relatedness satisfaction (RelS), and relatedness frustration (RelF). Responses are provided on a 5-point Likert scale ranging from 1 (strongly disagree) to 5 (strongly agree). Higher scores indicate greater levels of satisfaction or frustration within the corresponding psychological need domain. Internal consistency coefficients (Cronbach’s *α*) for the BPNSS subscales were as follows: AutoS = 0.92, AutoF = 0.83, CompS = 0.95, CompF = 0.94, RelS = 0.76, RelF = 0.91.

### Demographic questionnaire

Participants completed a demographic questionnaire developed by the researchers. Information regarding age, sex, body mass, height, marital status, training frequency, session duration, HIFT experience, perceived fatigue, perceived enjoyment, and participation in additional sports activities was collected.

### Procedure

Following ethical approval, permission was obtained from the managers of the participating fitness centers offering HIFT programs. Potential participants were informed about the aims and procedures of the study and provided written informed consent prior to participation. Data collection was conducted face-to-face immediately after regularly scheduled HIFT training sessions in designated recovery areas within the facilities. Participants completed the questionnaire package individually, which required approximately 15–20 min to complete. All responses were collected anonymously, and confidentiality was maintained throughout the study in accordance with ethical research standards.

### Statistical analysis

All statistical analyses were performed using IBM SPSS Statistics version 22.0 (IBM Corp. Armonk, NY, USA). Descriptive statistics are presented as means and standard deviations for continuous variables and frequencies and percentages for categorical variables. Normality assumptions were evaluated using Shapiro–Wilk tests together with skewness and kurtosis coefficients. Although some subscale scores deviated from normality, skewness and kurtosis values remained within acceptable limits (±2), supporting the use of parametric procedures. Independent-samples *t*-tests and one-way analyses of variance (ANOVA) were used to examine differences across demographic and training-related variables. Significant ANOVA findings were followed by least significant difference (LSD) *post hoc* comparisons. Pearson correlation coefficients were calculated to examine associations among study variables. Effect sizes were calculated for all group comparisons using Cohen’s *d* (small: 0.20, moderate: 0.50, large: 0.80; Cohen, 1988). To identify predictors of sport injury anxiety, a standard multiple linear regression analysis was performed. Predictors were selected based on theoretical relevance and prior empirical evidence. Age, sex, marital status, BMI, and the three psychological need frustration dimensions (autonomy frustration, competence frustration, and relatedness frustration) were entered simultaneously into the model. Statistical significance was established at *p* < 0.05. Prior to regression analysis, multicollinearity diagnostics indicated no violations, with all variance inflation factor (VIF) values below 3.00 (range: 1.17–2.59) and tolerance values above 0.10 (range: 0.39–0.86). The Durbin-Watson statistic (*d* = 2.03) confirmed the absence of residual autocorrelation. Residual normality was verified via Shapiro–Wilk test (*p* = 0.661).

## Results

Descriptive statistics for the subscales of the Sport Injury Anxiety Scale (SIAS) and the Basic Psychological Needs in Sport Scale (BPNSS) are presented in Table 1. Among the SIAS dimensions, reinjury anxiety demonstrated the highest mean score (*M* = 3.02, SD = 1.09), whereas loss of social support anxiety showed the lowest mean score (*M* = 1.48, SD = 0.69). The overall SIAS score was 2.23 (SD = 0.59). Regarding the BPNSS dimensions, the need satisfaction subscales yielded relatively high mean scores (autonomy satisfaction: *M* = 4.07; competence satisfaction: *M* = 4.11; relatedness satisfaction: *M* = 3.45), whereas the need frustration subscales were characterized by lower scores (autonomy frustration: *M* = 2.18; competence frustration: *M* = 1.75; relatedness frustration: *M* = 1.84).

**Table 1:** Descriptive statistics for SIAS and BPNSS subscales (*N* = 100).

Subscale	*M*	SD	Min	Max	Skew	Kurt
SIAS—LA	2.23	0.83	1.00	4.33	0.41	−0.22
SIAS—PW	1.52	0.68	1.00	4.67	1.88	5.17
SIAS—PA	2.92	1.09	1.00	5.00	0.08	−0.74
SIAS—DA	1.97	1.04	1.00	5.00	1.00	0.26
SIAS—LSS	1.48	0.69	1.00	4.33	1.72	3.53
SIAS—RA	3.02	1.09	1.00	5.00	−0.12	−0.71
SIAS total	2.23	0.59	1.00	3.74	0.36	0.12
BPNSS—AutoS	4.07	0.91	1.00	5.00	−1.67	3.30
BPNSS—AutoF	2.18	0.90	1.00	5.00	0.66	0.33
BPNSS—CompS	4.11	0.96	1.00	5.00	−1.55	2.44
BPNSS—CompF	1.75	0.92	1.00	5.00	1.47	1.88
BPNSS—RelS	3.45	0.86	1.00	5.00	−0.70	0.68
BPNSS—RelF	1.84	0.91	1.00	5.00	1.34	1.57

M, Mean; SD, Standard deviation; Skew, Skewness; Kurt, Kurtosis; SIAS, Subscales; LA, Loss of athletic ability; PW, Perceived weakness; PA, Pain anxiety; DA, Disappointment anxiety; LSS, Loss of social support; RA, Reinjury anxiety. BPNSS subscales: AutoS, Autonomy satisfaction; AutoF, Autonomy frustration; CompS, Competence satisfaction; CompF, Competence frustration; RelS, Relatedness satisfaction; RelF, Relatedness frustration.

Results of independent-samples *t*-tests comparing SIAS and BPNSS subscale scores by sex, together with Cohen’s *d* effect size estimates, are presented in Table 2. Analyses revealed a statistically significant sex difference only on the pain anxiety subscale of the SIAS [*t*(98) = −2.07, *p* = 0.041, *d* = −0.44]. Women reported significantly higher pain anxiety scores (*M* = 3.23, SD = 0.94) than men (*M* = 2.76, SD = 1.13). The magnitude of this difference was classified as moderate according to conventional benchmarks (Cohen, 1988). The SIAS total score showed a borderline non-significant trend favoring women [*t*(98) = −1.73, *p* = 0.087, *d* = −0.37], which did not reach the conventional significance threshold of *p* < 0.05. No significant sex differences were observed for any BPNSS subscale (all *p*s > 0.05).

**Table 2:** Independent-samples *t*-test results comparing SIAS and BPNSS scores by sex.

Subscale	*M* (men)	SD	*M* (women)	SD	*t*(98)	*p*	*d*	Effect
SIAS—LA	2.15	0.80	2.39	0.87	−1.41	0.161	−0.30	Small
SIAS—PW	1.52	0.63	1.53	0.79	−0.06	0.949	−0.01	–
SIAS—PA^*^	2.76	1.13	3.23	0.94	−2.07	0.041	−0.44	Moderate
SIAS—DA	1.94	1.02	2.02	1.10	−0.34	0.734	−0.07	–
SIAS—LSS	1.49	0.70	1.46	0.69	0.16	0.870	0.04	–
SIAS—RA	2.87	1.13	3.31	0.96	−1.93	0.056	−0.41	Small
SIAS total	2.16	0.62	2.38	0.51	−1.73	0.087	−0.37	Small
BPNSS—AutoS	4.10	0.90	4.02	0.96	0.43	0.672	0.09	–
BPNSS—AutoF	2.22	0.95	2.10	0.79	0.65	0.515	0.14	–
BPNSS—CompS	4.16	0.93	4.01	1.02	0.75	0.458	0.16	–
BPNSS—CompF	1.73	0.93	1.78	0.91	−0.25	0.805	−0.05	–
BPNSS—RelS	3.43	0.85	3.48	0.90	−0.27	0.789	−0.06	–
BPNSS—RelF	1.91	0.99	1.71	0.74	1.06	0.294	0.22	Small

Men *n* = 66; Women *n* = 34. *d* = Cohen’s d effect size (small: 0.20, moderate: 0.50, large: 0.80). ^*^p < 0.05.

**Figure 1:**
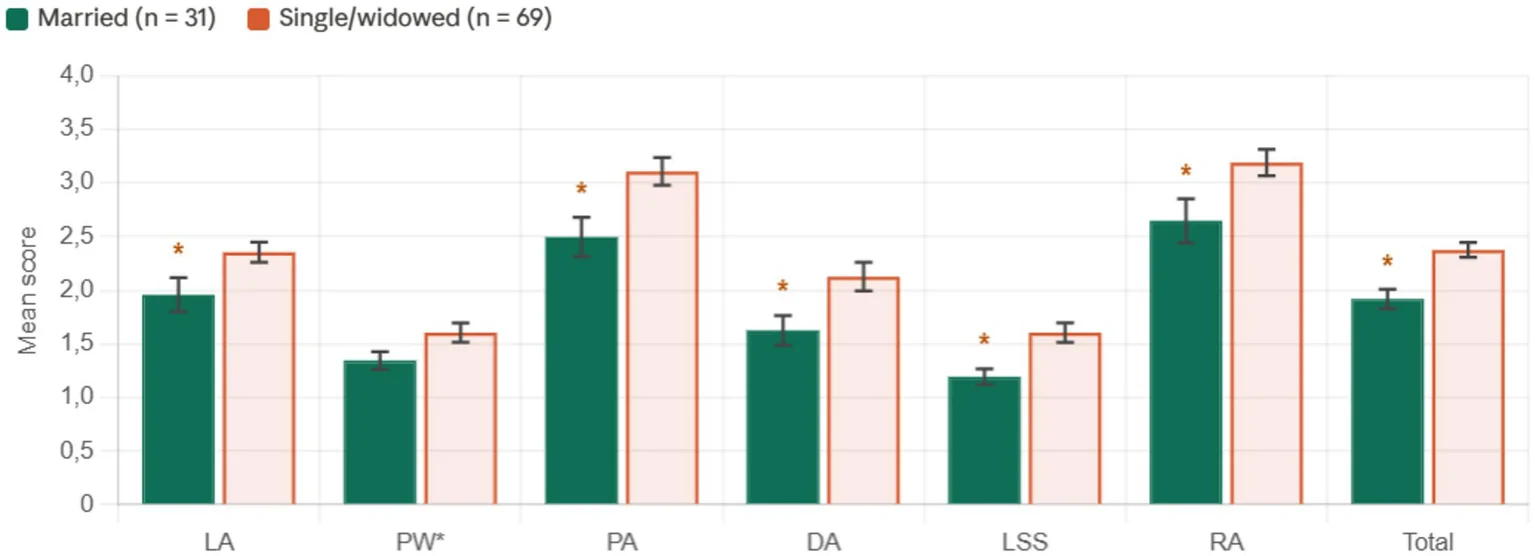
Mean sport injury anxiety subscale scores by marital status with standard error bars. LA, Loss of athletic ability; PW, Perceived weakness; PA, Pain anxiety; DA, Disappointment anxiety; LSS, Loss of social support; RA, Reinjury anxiety. Error bars represent ±1 standard error of the mean. Married group *n* = 31; Single/widowed group *n* = 69. Asterisks (^*^) indicate statistically significant between-group differences (*p* < 0.05, independent-samples *t*-test). Effect sizes ranged from moderate to large (Cohen’s *d* = 0.49–0.83).

For marital status comparisons, married participants were contrasted with unmarried/widowed participants. Married participants reported significantly lower total sport injury anxiety than unmarried participants, with a large effect size. Significant differences were also observed for most injury anxiety subdimensions, indicating consistently lower anxiety levels among married athletes. In contrast, psychological need satisfaction and frustration scores did not differ according to marital status (Table 3; Figure 1).

**Table 3:** Independent-samples *t*-test results comparing SIAS and BPNSS scores by marital status.

Subscale	*M* (married)	SD	*M* (single)	SD	*t*(98)	*p*	*d*	Effect
SIAS—LA^*^	1.96	0.89	2.35	0.78	−2.25	0.026	−0.49	Moderate
SIAS—PW	1.34	0.46	1.60	0.75	−1.79	0.077	−0.39	Small
SIAS—PA^*^	2.50	1.02	3.11	1.07	−2.68	0.009	−0.58	Moderate
SIAS—DA^*^	1.62	0.77	2.13	1.11	−2.28	0.025	−0.49	Moderate
SIAS—LSS^*^	1.19	0.40	1.60	0.76	−2.84	0.006	−0.61	Moderate
SIAS—RA^*^	2.65	1.15	3.19	1.03	−2.36	0.020	−0.51	Moderate
SIAS total^**^	1.92	0.51	2.38	0.58	−3.81	< 0.001	−0.83	Large
BPNSS—AutoS	4.04	1.10	4.09	0.83	−0.24	0.809	−0.05	–
BPNSS—AutoF	2.03	1.05	2.24	0.82	−1.09	0.280	−0.24	–
BPNSS—CompS	3.99	1.11	4.17	0.89	−0.82	0.412	−0.18	–
BPNSS—CompF	1.52	0.94	1.85	0.90	−1.70	0.092	−0.37	Small
BPNSS—RelS	3.35	1.02	3.50	0.78	−0.79	0.431	−0.17	–
BPNSS—RelF	1.73	0.98	1.89	0.88	−0.81	0.418	−0.18	–

Married *n* = 31; Single/widowed *n* = 69. *d* = Cohen’s *d* effect size (small: 0.20, moderate: 0.50, large: 0.80). ^*^p < 0.05; ^**^p < 0.001.

One-way analyses of variance revealed significant differences across training frequency groups for loss of athletic ability anxiety, autonomy frustration, and relatedness frustration (Table 4). *Post hoc* comparisons showed that participants training more than 4 days per week generally reported lower injury-related concerns and lower levels of psychological need frustration than participants training less frequently.

**Table 4:** One-way ANOVA results for SIAS and BPNSS subscales by weekly training frequency.

Subscale	2 d/wk	3 d/wk	4 d/wk	4 + d/wk	*F*(3, 96)	*p*	Post-hoc
SIAS—LA^*^	2.31	2.42	2.39	1.83	3.23	0.026	3 d > 4 + d; 4 d > 4 + d
SIAS total	2.42	2.33	2.24	2.01	2.20	0.094	–
BPNSS—AutoF^*^	2.67	2.27	2.15	1.83	3.22	0.026	2 d > 4 + d; 2 d > 4 d
BPNSS—RelS	3.27	3.18	3.62	3.71	2.64	0.054	–
BPNSS—RelF^*^	2.13	2.11	1.72	1.49	3.17	0.028	2 d > 4 + d; 3 d > 4 + d

2 d/wk = 2 days per week (*n* = 15); 3 d/wk = 3 days per week (*n* = 32); 4 d/wk = 4 days per week (*n* = 25); 4 + d/wk = More than 4 days per week (*n* = 28). Post-hoc column: ‘>’ indicates the group with the higher mean score. ^*^p < 0.05.

**Figure 2:**
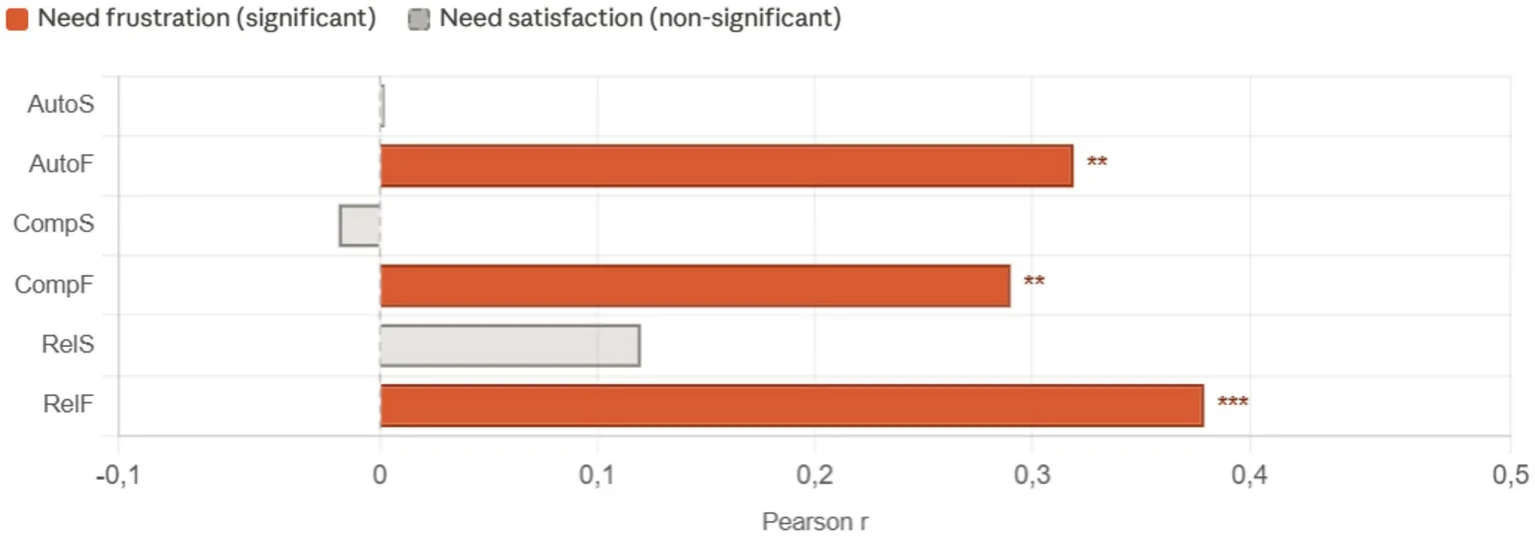
Pearson correlations between total sport injury anxiety (SIAS) and psychological need satisfaction and frustration subscales. AutoS, Autonomy satisfaction; AutoF, Autonomy frustration; CompS, Competence satisfaction; CompF, Competence frustration; RelS, Relatedness satisfaction; RelF, Relatedness frustration. Filled bars (orange) represent need frustration subscales; open bars (gray) represent need satisfaction subscales. The dashed vertical line indicates *r* = 0. Need frustration subscales demonstrated significant positive associations with sport injury anxiety, whereas need satisfaction subscales showed no significant associations (all *p*s > 0.05). ^**^p < 0.01; ^***^p < 0.001.

Pearson correlation coefficients are presented in Table 5. Significant positive associations were observed between total sport injury anxiety and autonomy frustration, competence frustration, and relatedness frustration. In contrast, no significant relationships were found between total sport injury anxiety and any of the psychological need satisfaction dimensions. Among the frustration variables, relatedness frustration demonstrated the strongest correlation with injury anxiety (Figure 2).

**Table 5:** Pearson correlation coefficients among SIAS and BPNSS subscales.

	SIAS total	LA	PW	PA	DA	LAA	RA	AutoS	AutoF	CompS	CompF	RelF
1. SIAS total	–	0.67^**^	0.60^**^	0.57^**^	0.65^**^	0.67^**^	0.71^**^	0.00	0.32^**^	−0.02	0.29^**^	0.38^**^
2. LA		–	0.35^**^	0.27^**^	0.27^**^	0.39^**^	0.43^**^	−0.07	0.14	−0.08	0.16	0.22^*^
3. PW			–	0.16	0.44^**^	0.75^**^	0.09	−0.35^**^	0.46^**^	−0.37^**^	0.41^**^	0.38^**^
4. PA				–	0.12	0.13	0.35^**^	0.22^*^	0.13	0.21^*^	0.06	0.18
5. DA					–	0.50^**^	0.27^**^	−0.08	0.32^**^	−0.08	0.33^**^	0.36^**^
6. LSS						–	0.22^*^	−0.23^*^	0.42^**^	−0.20^*^	0.38^**^	0.42^**^
7. RA							–	0.21^*^	0.01	0.17	0.00	0.08
8. AutoS								–	−0.05	0.86^**^	−0.11	−0.09
9. AutoF									–	−0.11	0.47^**^	0.67^**^
10. CompS										–	−0.23^*^	−0.12
11. CompF											–	0.66^**^
12. RelF												–

1 = SIAS Total; 2 = LA; 3 = PW; 4 = PA; 5 = DA; 6 = LSS; 7 = RA; 8 = AutoS; 9 = AutoF; 10 = CompS; 11 = CompF; 12 = RelF. Lower triangle omitted to avoid redundancy. ^*^p < 0.05. ^**^p < 0.01.

**Figure 3:**
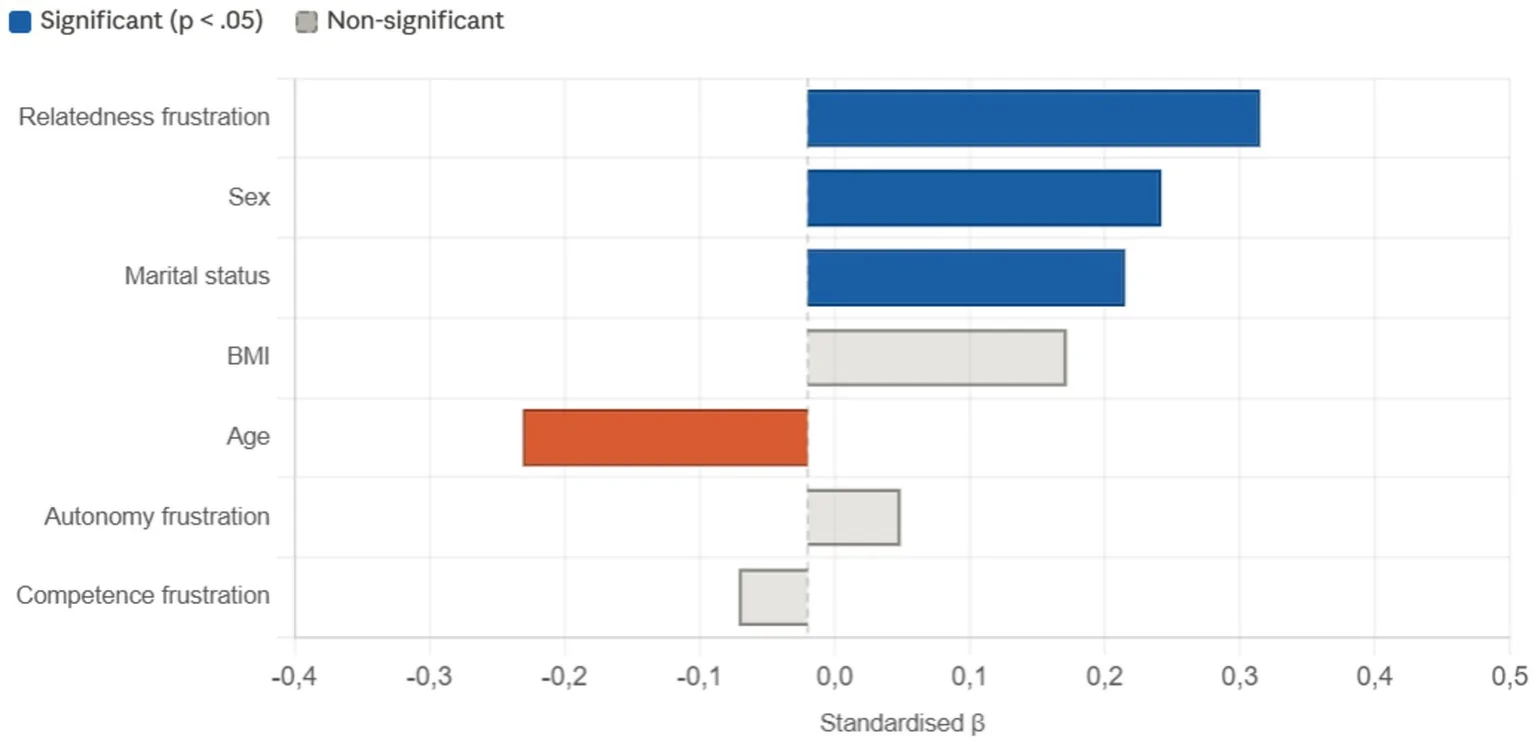
Standardized regression coefficients (*β*) for predictors of total sport injury anxiety (SIAS). Blue bars = significant positive predictors (*p* < 0.05); orange bar = significant negative predictor (*p* < 0.05); gray bars = non-significant predictors (*p* > 0.05). Sex coded as 1 = men, 2 = women; marital status coded as 1 = married, 2 = single/widowed. The dashed vertical line indicates *β* = 0. Model fit: *R*^2^ = 0.318, adjusted *R*^2^ = 0.267, *F*(7, 92) = 6.14, *p* < 0.001. Among the psychological need frustration dimensions, only relatedness frustration emerged as a significant independent predictor (*β* = 0.34, *p* = 0.017); autonomy frustration (*β* = 0.07, *p* = 0.558) and competence frustration (*β* = −0.05, *p* = 0.668) did not contribute significantly when all predictors were entered simultaneously.

**Figure 4:**
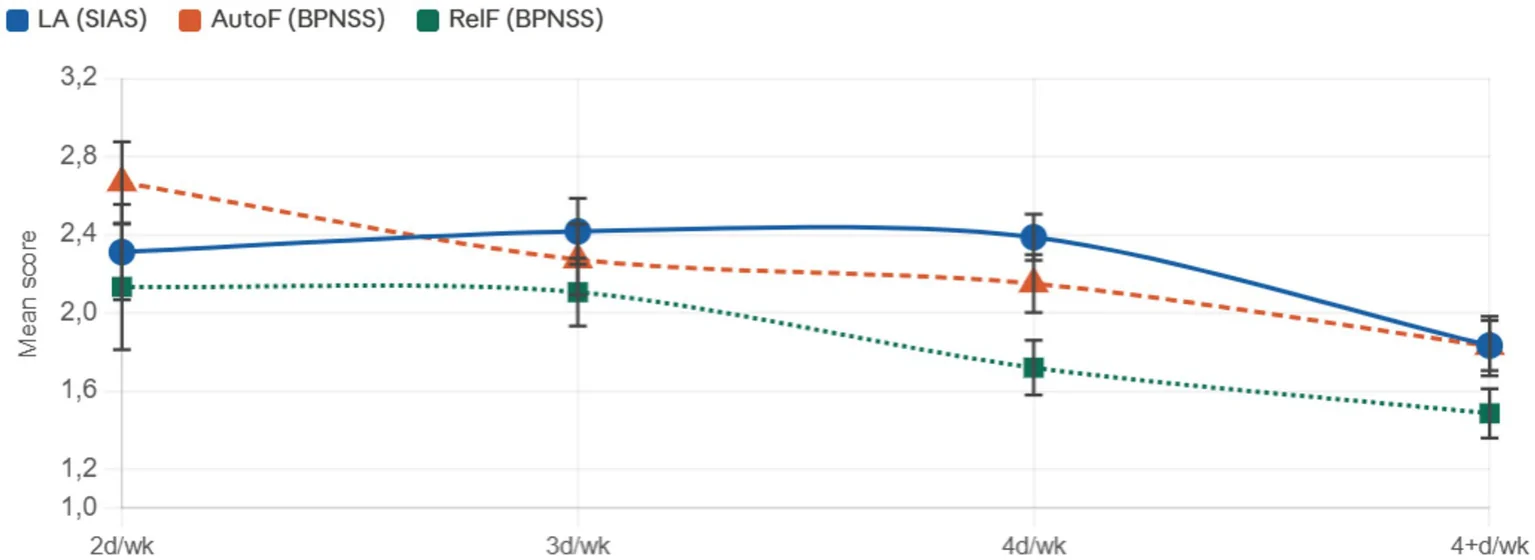
Mean scores for loss of athletic ability anxiety (LA), autonomy frustration (AutoF), and relatedness frustration (RelF) as a function of weekly HIFT training frequency. LA, Loss of athletic ability (SIAS); AutoF, Autonomy frustration (BPNSS); RelF, Relatedness frustration (BPNSS). Error bars represent ±1 standard error of the mean. Circle markers with solid line = LA; triangle markers with dashed line = AutoF; square markers with dotted line = RelF. 2 d/wk. = 2 days per week (*n* = 15); 3 d/wk. = 3 days per week (*n* = 32); 4 d/wk. = 4 days per week (*n* = 25); 4 + d/wk. = four or more days per week (*n* = 28). All three variables demonstrated a consistent declining trend with increasing training frequency. Significant group differences were identified for LA [*F*(3, 96) = 3.23, *p* = 0.026], AutoF [*F*(3, 96) = 3.22, *p* = 0.026], and RelF [*F*(3, 96) = 3.17, *p* = 0.028] via one-way ANOVA with LSD *post hoc* comparisons.

To identify independent predictors of sport injury anxiety, a standard multiple linear regression analysis was conducted with total SIAS as the criterion variable. Age, BMI, sex, marital status, and the three psychological need frustration dimensions were entered simultaneously as predictors. Results are presented in Table 6. The regression model was statistically significant, *F*(7, 92) = 6.14, *p* < 0.001, explaining 31.8% of the variance in sport injury anxiety (*R*^2^ = 0.318, adjusted *R*^2^ = 0.267). Age, sex, marital status, and relatedness frustration emerged as significant predictors. Among the psychological need frustration dimensions, only relatedness frustration remained a significant independent predictor when all variables were considered simultaneously (Figures 3, 4).

**Table 6:** Multiple linear regression predicting total SIAS scores.

Predictor	*B*	SE	*β*	*t*	*p*
Age	−0.02	0.01	−0.21	−2.12	0.037^*^
BMI	0.03	0.02	0.19	1.80	0.074
Sex (1 = men, 2 = women)	0.33	0.13	0.26	2.61	0.011^*^
Marital status (1 = married, 2 = single)	0.28	0.11	0.24	2.53	0.013^*^
Autonomy frustration	0.05	0.08	0.07	0.59	0.558
Competence frustration	−0.03	0.08	−0.05	−0.43	0.668
Relatedness frustration^*^	0.22	0.09	0.34	2.42	0.017^*^

*N* = 100. *B* = Unstandardised regression coefficient; SE = Standard error; *β* = Standardized regression coefficient. Sex coded as 1 = men, 2 = women. Marital status coded as 1 = married, 2 = single/widowed. Model: *R*^2^ = 0.318, adjusted *R*^2^ = 0.267, *F*(7, 92) = 6.14, *p* < 0.001. ^*^p < 0.05.

## Discussion

The present study examined the demographic and psychological factors associated with sport injury anxiety among High-Intensity Functional Training (HIFT) participants, with particular emphasis on the role of psychological need frustration within the framework of Self-Determination Theory (SDT). Overall, the findings supported the study hypotheses and provided novel evidence regarding the psychological correlates of injury-related anxiety in HIFT settings. Psychological need frustration was positively associated with sport injury anxiety, whereas psychological need satisfaction demonstrated no significant relationship with injury-related concerns. Furthermore, age, sex, and marital status emerged as significant predictors of sport injury anxiety, highlighting the contribution of both psychological and demographic factors to injury-related concerns among recreational HIFT participants.

The overall level of sport injury anxiety in the present sample was relatively low (*M* = 2.23, SD = 0.59), falling below the midpoint of the scale and slightly lower than values previously reported in heterogeneous athletic populations (Tanyeri, 2019). This pattern may reflect several characteristics of High-Intensity Functional Training (HIFT) environments, including strong social cohesion, supportive coaching practices, and opportunities for continuous performance development (Partridge et al., 2014). At the subscale level, reinjury anxiety demonstrated the highest mean score, whereas loss of social support anxiety showed the lowest. Consistent with previous research, concerns regarding future injuries may be associated with the cumulative physical demands and perceived injury risk inherent in high-intensity training modalities (Hak et al., 2013; Montalvo et al., 2017). Conversely, the strong sense of community and social connectedness commonly reported in HIFT settings may help explain the relatively low levels of anxiety related to loss of social support (Heinrich et al., 2014).

Regarding psychological needs, participants reported relatively high levels of need satisfaction and comparatively low levels of need frustration. This pattern may indicate that the High-Intensity Functional Training (HIFT) environment was generally perceived as supportive of basic psychological needs, which is consistent with previous research linking need-supportive exercise and sport settings to greater motivation, engagement, and sustained participation (Ryan and Deci, 2017; Árok and Szabo, 2025). Indeed, previous studies have suggested that HIFT programs often foster a strong sense of community and interpersonal connection, which may enhance participants’ feelings of belongingness and social integration (Dominski et al., 2021).

The most important finding of the present study was that psychological need frustration, rather than need satisfaction, was associated with sport injury anxiety. All three need frustration dimensions demonstrated significant positive associations with injury anxiety, whereas need satisfaction showed no significant relationships with any SIAS subscale. These findings are consistent with the “dark side” perspective of Self-Determination Theory, which proposes that need frustration represents a distinct psychological process more strongly associated with maladaptive outcomes than the mere absence of need satisfaction (Alexe et al., 2022; Cansler et al., 2023). Among the three need frustration dimensions, only relatedness frustration emerged as an independent predictor of injury anxiety in the regression model. Given that HIFT programs are typically characterized by group-based participation, shared training experiences, and strong social interaction, individuals who perceive themselves as socially disconnected or insufficiently supported within their training environment may be particularly vulnerable to injury-related concerns (Dominski et al., 2021). Furthermore, social connectedness has been shown to negatively predict anxiety among physically active individuals and athletes, suggesting that a diminished sense of belonging may amplify anxiety responses in exercise and sport settings (Soares et al., 2022).

With regard to sex differences, female participants reported higher levels of pain-related anxiety than male participants, although no significant differences were observed for overall sport injury anxiety. This finding is partially consistent with previous research indicating greater injury-related concerns among female athletes (Correia and Rosado, 2019; John et al., 2025). One possible explanation may involve sex-related differences in pain perception and pain catastrophizing, as women often report greater sensitivity to pain-related experiences (Bartley and Fillingim, 2013). However, the absence of broader sex differences suggests that the relationship between sex and injury anxiety may be context-dependent and influenced by the specific characteristics of the training environment.

A particularly noteworthy finding was the large effect of marital status on injury anxiety (*d* = 0.83), with married participants reporting consistently lower scores across nearly all injury anxiety dimensions. Previous studies have shown that married individuals tend to experience lower levels of perceived stress and psychological overload compared with individuals in other relationship statuses (Amirkhan and Vandenbelt, 2023; Bartley and Fillingim, 2013). The emotional, social, and practical support provided within a marital relationship may serve as a protective factor against injury-related concerns by enhancing psychological security and coping resources, consistent with the stress-buffering hypothesis (Simons and Bird, 2023).

Training frequency also demonstrated meaningful associations with psychological outcomes. Participants who trained more frequently reported lower levels of autonomy frustration, relatedness frustration, and concerns regarding loss of athletic ability. These findings may reflect greater technical proficiency, self-efficacy, physical adaptation, and stronger social integration within HIFT training communities among highly engaged participants (Kliszczewicz et al., 2014). Regular participation may also facilitate the development of supportive interpersonal relationships and a stronger sense of belonging, thereby reducing both psychological need frustration and injury-related concerns. Nevertheless, given the cross-sectional nature of the present study, the direction of these relationships cannot be determined. It is equally plausible that individuals with lower injury anxiety and better psychological functioning are more likely to sustain higher levels of training participation. Therefore, longitudinal studies are needed to clarify the causal mechanisms underlying these associations.

The regression model identified age as a significant negative predictor of sport injury anxiety (*β* = −0.21, *p* = 0.037), indicating that injury-related concerns tended to decrease with increasing age. This finding is consistent with the observed negative correlation between age and injury anxiety (*r* = −0.28, *p* = 0.005) and may reflect greater training experience, more realistic perceptions of injury risk, and age-related reductions in general anxiety (Correia and Rosado, 2019). As individuals accumulate training experience over time, they may develop greater confidence in their physical capabilities, more effective coping strategies, and a more balanced understanding of injury risk, which may contribute to lower levels of injury-related anxiety. Consequently, younger and less experienced HIFT participants may constitute a subgroup particularly vulnerable to injury-related concerns and may therefore benefit from targeted educational and psychological support strategies aimed at improving confidence, risk perception, and coping skills.

Limitations and future directions: Several limitations should be considered when interpreting the findings of the present study. First, the cross-sectional design precludes any conclusions regarding causality between psychological need frustration and sport injury anxiety. Second, all variables were assessed using self-report instruments, which may be susceptible to social desirability, response style, and recall biases. Third, participants were recruited from fitness centers offering High-Intensity Functional Training (HIFT) programs within a single metropolitan area using a convenience sampling strategy, which may limit the generalizability of the findings to other populations and training contexts. Finally, the relatively small number of divorced and widowed participants prevented a more detailed examination of differences according to relationship status. Additionally, information regarding participants’ prior sport-related injury history (i.e., presence versus absence of previous injuries, and single versus recurrent injury episodes) was not collected in the present study. Given that injury history is a well-established antecedent of injury-related anxiety, its absence limits the extent to which the observed associations between psychological need frustration and sport injury anxiety can be interpreted independently of participants’ injury background. Future studies should assess and control for injury history to clarify its potential contribution to sport injury anxiety in HIFT populations. To evaluate the potential influence of common method bias, Harman’s single-factor test was performed by entering all questionnaire items into an unrotated principal component analysis. The first factor accounted for 26.78% of the total variance, which is substantially below the recommended threshold of 50%, suggesting that common method bias was unlikely to have materially affected the results (Podsakoff et al., 2003).

From an applied perspective, the findings suggest that HIFT coaches, instructors, and practitioners may benefit from fostering socially supportive and inclusive training environments that minimize experiences of relatedness frustration. Practical strategies may include encouraging peer support, promoting cooperative rather than excessively competitive interactions, reducing maladaptive social comparison, and ensuring that participants of all performance levels feel respected, accepted, and valued within the training community. Furthermore, given that younger and unmarried participants demonstrated higher levels of injury-related anxiety, targeted psychoeducational interventions and psychological support programs may be particularly beneficial for these groups.

Future research should employ longitudinal, prospective, and experimental designs to clarify the causal relationships between psychological need frustration and sport injury anxiety. Advanced analytical approaches, such as structural equation modeling, may provide further insight into the psychological mechanisms linking need frustration to injury-related concerns. Incorporating detailed injury history, including injury frequency and chronicity, as a covariate or moderator would also help disentangle its relative contribution to injury-related anxiety from that of psychological need frustration. In addition, intervention studies examining the effectiveness of need-supportive coaching behaviors and socially inclusive training practices may offer valuable practical implications for reducing injury anxiety. Cross-cultural investigations and comparisons across different exercise modalities may further enhance understanding of the contextual factors that influence injury-related anxiety in high-intensity training environments.

## Conclusion

The present study examined sport injury anxiety among High-Intensity Functional Training (HIFT) participants from the perspective of Self-Determination Theory, with particular emphasis on psychological need frustration. The findings revealed that psychological need frustration, rather than psychological need satisfaction, was significantly associated with sport injury anxiety. Among the three need frustration dimensions, relatedness frustration emerged as the strongest psychological correlate and a significant independent predictor of injury-related anxiety. In addition to psychological factors, several demographic characteristics contributed to the explanation of injury anxiety. Female participants reported higher levels of pain-related anxiety, younger individuals demonstrated greater injury-related concerns, and married participants exhibited lower anxiety levels than their unmarried counterparts. Taken together, these findings suggest that both psychological and social factors play an important role in shaping injury-related concerns among recreational HIFT participants. The regression model explained 31.8% of the variance in sport injury anxiety, indicating that psychological need frustration and selected demographic variables contribute meaningfully to understanding injury-related concerns in this population. These findings extend previous research grounded in Self-Determination Theory by highlighting the importance of psychological need frustration, particularly relatedness frustration, in the context of sport injury anxiety. From an applied perspective, coaches, instructors, and practitioners involved in HIFT programs may benefit from creating training environments that promote social inclusion, strengthen interpersonal connections, and reduce experiences of psychological need frustration. Encouraging supportive peer interactions and fostering a strong sense of belonging may help mitigate injury-related concerns among participants. By demonstrating that psychological need frustration—and relatedness frustration in particular—constitutes a meaningful predictor of sport injury anxiety, this study extends the application of Self-Determination Theory to a relatively underexplored area of sport and exercise psychology and provides a foundation for future research and evidence-based practice in HIFT settings.

## Data Availability

The raw data supporting the conclusions of this article will be made available by the authors, without undue reservation.
